# A rapid smartphone-based lactate dehydrogenase test for neonatal diagnostics at the point of care

**DOI:** 10.1038/s41598-019-45606-0

**Published:** 2019-06-26

**Authors:** Cecilia Pegelow Halvorsen, Linus Olson, Ana Catarina Araújo, Mathias Karlsson, Trang Thị Nguyễn, Dung T. K. Khu, Ha T. T. Le, Hoa T. B. Nguyễn, Birger Winbladh, Aman Russom

**Affiliations:** 1 Department of clinical research and education, Södersjukhuset, Karolinska Institutet, Stockholm, Sweden; 2 Neonatal Unit at Sachs’ Children and Youth hospital, Stockholm, Sweden; 3 Department of Women’s and Children’s Health, Karolinska Institutet, Stockholm, Sweden; 4 Department of Public Health Sciences, Karolinska Institutet, Stockholm, Sweden; 5 Training and Research Academic Collaboration (TRAC) Sweden - Vietnam, Hanoi, Vietnam; 6 Calmark Sweden AB, Stockholm, Sweden; 7 Department of Medical Sciences, Biomedical Structure and Function, Uppsala University, Uppsala, Sweden; 8 Neonatal Intensive Care Unit, Vietnam National Children’s Hospital, Hanoi, Vietnam; 9 Research Institute for Child Health, Hanoi, Vietnam; 10 Division of Nanobiotechnology, Department of Protein Science, Science for Life Laboratory, School of Engineering Sciences in Chemistry, Biotechnology and Health, KTH Royal Institute of Technology, Stockholm, Sweden

**Keywords:** Diseases, Diagnosis, Biomedical engineering

## Abstract

There is a growing recognition of the importance of point-of-care tests (POCTs) for detecting critical neonatal illnesses to reduce the mortality rate in newborns, especially in low-income countries, which account for 98 percent of reported neonatal deaths. Lactate dehydrogenase (LDH) is a marker of cellular damage as a result of hypoxia-ischemia in affected organs. Here, we describe and test a POC LDH test direct from whole blood to provide early indication of serious illness in the neonate. The sample-in-result-out POC platform is specifically designed to meet the needs at resource-limited settings. Plasma is separated from whole blood on filter paper with dried-down reagents for colorimetric reaction, combined with software for analysis using a smartphone. The method was clinically tested in newborns in two different settings. In a clinical cohort of newborns of Stockholm (n = 62) and Hanoi (n = 26), the value of R using Pearson’s correlation test was 0.91 (p < 0.01) and the R^2^ = 0.83 between the two methods. The mean LDH (±SD) for the reference method vs. the POC-LDH was 551 (±280) U/L and 552 (±249) U/L respectively, indicating the clinical value of LDH values measured in minutes with the POC was comparable with standardized laboratory analyses.

## Introduction

Every day approximately 15 000 children die before their fifth birthday, 46% of them die within the first 28 days of life, according to statistics of a WHO report from 2017^1^. Neonatal mortality has been the greatest hindrance to achieving the Millennium Development Goal 4 stated by the United Nations in 2000^2^. The problem of neonatal mortality is especially severe in low-income countries, which have 98% of the reported neonatal deaths. The most important causes of neonatal mortality have been described as complications associated with preterm birth, birth asphyxia and/or infections^3^. In addition to availability of healthcare workers with necessary skills for diagnose and treatment of complications or illnesses, there also have to be appropriate resources^4^. Consequently, there has been growing recognition of the importance of point-of-care tests (POCTs) for detecting the most critical neonatal illnesses to reduce the mortality rate in newborns. When coupled with effective treatment strategies, low-cost POC diagnostics that can be administered in low-resource settings have the potential to reduce neonatal mortality^5^. However, to date, robust, affordable and technically simple methods to measure critical neonatal illness remain unavailable.

Lactate dehydrogenase (LDH), as a marker of cellular damage as a result of hypoxia-ischemia in affected organs in the neonate is well known. LDH is present in all human cells and may be a good predictive biomarker for adverse neurodevelopmental outcome after perinatal asphyxia^6,7^. Substantial increases in LDH compared to controls and mild cases have been reproducibly reported in newborns suffering from severe asphyxia and other conditions in need of intensive care, suggesting LDH may serve as a robust biomarker for severe disease. LDH has also been reported to be elevated in newborns in need of neonatal intensive care (NICU)^8^ and other neonatal diseases e.g., transient tachypnea^9^ and necrotizing enterocolitis^10^. Existing technologies to perform blood analyses are either time consuming or the instrument and technical operation is too demanding to be applied at the POC.

Among the many new emerging technologies, microfluidic lab-on-a-chip devices have the potential to overcome these limitations. The most attractive features of microscale approaches for blood analysis include the need for only microliter blood volumes obtainable without venipuncture, and simple operation by minimally trained personnel. These features are particularly essential for diagnostics that can have an impact on neonatal diagnostics. However, for blood diagnostics, the integration of several distinct components and steps is extremely challenging. Ideally, for resource-limited settings, POCT should meet the ASSURED (affordable, sensitive, specific, user-friendly, robust & rapid, equipment-free, and deliverable) criteria^11,12^. Paper-based lateral flow assays (LFAs) are able to fulfill all the ASSURED criteria. However, one of the main challenges of developing LFAs is in achieving clinically relevant sensitivity and specificity. Paper based^13^ and microfluidic rotational slipdisc based^14^ LDH tests have been reported using plasma as the input sample. The separation of plasma from whole blood is a major problem, especially in patient-groups with a broad range of hematocrit as in newborns. In the present work, we set out to develop a robust LDH-based POC diagnostics platform especially designed to meet the ASSURED criteria for use in newborns.

Here, we report on an early, manually manufactured, prototype of a rapid paper-based POC assay that requires minute amounts of whole blood as input and delivers colorimetric-based measurement of the LDH concentration in less than four minutes using a smartphone camera. The POC analytical device involved lateral separation of whole blood into blood plasma on a set of filter papers, colorimetric reaction on filter membranes using dried-down chemical reagents, and analysis of concentration using software on a smartphone. We assessed the suitability of the platform regarding the analytical and clinical performance in two different perinatal settings.

## Results

### Pre-clinical phase

**Figure 1:**
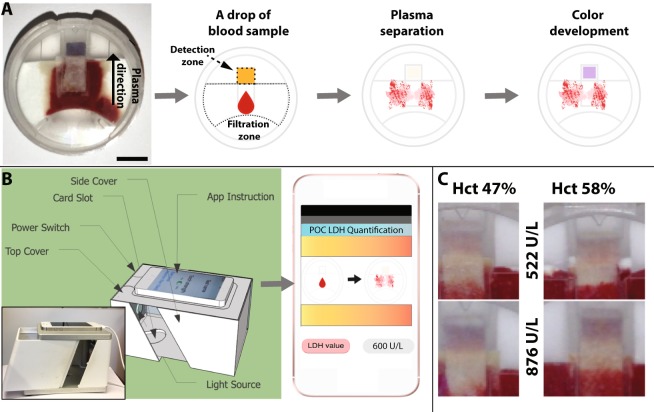
POC system for direct analysis of LDH from whole blood. (**A**) Overview of the POC device, which consists of a plastic cartridge that holds filter papers to separate plasma from whole blood samples and then expose the plasma to pre-dried reagents after separation for the colorimetric LDH assay. For clarity, the detection and blood filtration zone is highlighted. Scale bar: 0.4 cm. (**B**) The cartridge is placed on a designated slot inside a box, before moving the side cover to close the box. The box keeps the distance between the smartphone and the cartridge fixed, while helping to ensure similar light conditions between different batches. The built-in app on the smartphone provides a simple guide that leads the user through the assay process and captures an image of the cartridge after locating its position. The software on the smartphone directly identifies RGB values from the image and sends them to a text file linked to the system, along with the raw picture. The entire assay process takes approximately 3 minutes. (**C**) Blood plasma separation and colorimetric reaction for two different LDH levels and hematocrit concentrations. The POC device can handle samples with high levels of hematocrit (up to 58% tested), and it performs a hemolysis-free blood separation in less than 15 s.

The LDH-POC system consists of a disposable paper strip with dry reagents for the LDH assay mounted on an injection molded plastic cartridge, and a smartphone with a customized application (LDH app) for colorimetric detection of the LDH activity. An overview of the whole system is given in Fig. 1. Figure 1B shows the setup used to monitor the reaction in real time. A dedicated smartphone app captures a set of images every 15 seconds, converts the images into a text file and compiles the information together with the raw images into a file for storage. After approximately three minutes, the end-user receives the calculated LDH value on the screen. Figure 1C shows blood plasma filtration for two different haematocrit concentrations. The POC device was capable of performing haemolysis-free blood separation and colorimetric determination of LDH concentration at levels of haematocrit up to 58% of haematocrit tested, (see Supplementary Fig. 1).

**Figure 2:**
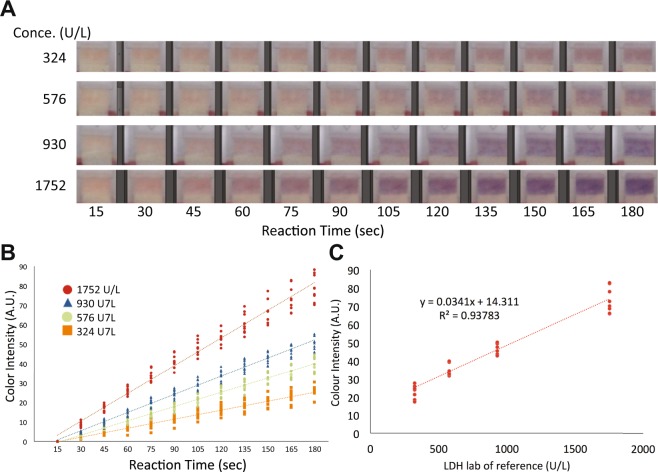
Characterization of the POC colorimetric LDH assay. (**A**) Image of the detection area of the POC device, showing color development over time for four different clinically relevant LDH concentrations. The color difference is clearly visible after one minute with the naked eye. (**B**) The color intensity (average red channel value) versus reaction time for the four different LDH concentrations. Each dot represents one LDH level/sample tested and the line indicates the average of 6–10 replicate runs for each sample. There is less color overlap for a reaction time of 120–165 seconds. (**C**) Correlation between the color intensity and LDH levels for a reaction time of 165 seconds.

Figure 2A shows zoomed in images of the detection area captured over a time period of three minutes. Each image was taken every 15 seconds, giving a total of 12 pictures to be analysed. The colour development depended on the reaction time. Important, there was a clear colour difference over time between the four clinically relevant LDH levels tested. We developed software in a smartphone app that analyses the RGB values in each image. The red channel was found to be the best for correlating the colour development with the LDH levels. Figure 2B shows the resulting average red channel values from each image taken every 15 seconds for three minutes. For a reaction time of 120–165 seconds, there was good colour contrast between the four LDH levels (1752 U/L, 930 U/L, 576 U/L and 324 U/L) with no overlapping and a good precision within the replicates. Figure 2C shows a plot of the normalized red channel values versus LDH levels for a reaction time of 165 sec. For this data, we obtained a correlation of R^2^ = 0.94.

**Figure 3:**
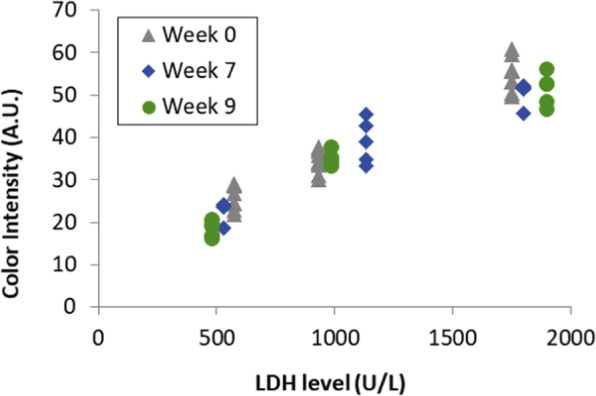
Shelf life test. Test of the POC colorimetric assay on adult blood at week 0 (grey dots), and after 7 (blue dots) and 9 (green dots) weeks. The color development as a result of the reactivity of the dry chemistry reagents is similar.

The shelf life test to make sure the reagents and components of the POC device were able to withstand shipping, handling and storage over a minimum period of two months is presented in Fig. 3. Storing the dry reagents over a period of nine weeks had almost no change on the reactivity of the chemistry. After thorough testing, we concluded that the POC platform was suitable to be further tested in newborn patients in clinical settings. The comparison of the two reference laboratories (Stockholm and Hanoi) showed a bias <10 percent for the controls used.

#### Clinical phase

**Figure 4:**
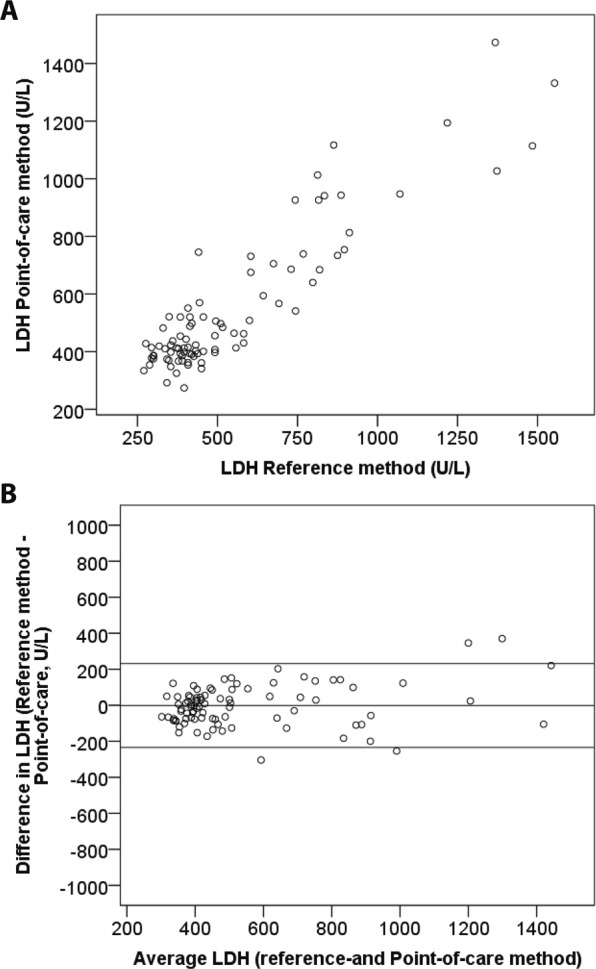
(**A**) Correlation between the point-of-care method for LDH measurement and the reference method. (**B**) A Bland-Altman plot showing the differences between the two methods (Y-axis) and the average results of the methods (X-axis).

Paired LDH result from both POC-LDH and reference method was obtained from 88 infants and thus included in the study (62 infants from Stockholm and 26 infants from Hanoi). The value of R using Pearson’s correlation test was 0.91 (p < 0.01) and the R^2^ = 0.83 between the two methods (Fig. 4A**)**. The mean LDH (±SD) for the samples of the 88 infants using the reference method vs. the POC-LDH was 551 (±280) U/L and 552 (±249) U/L respectively. The mean difference and limits of agreement between the reference methods and POC-LDH was 1 U/L (−233–231) and presented as a Bland-Altman plot in Fig. 4B. Among infants born at Stockholm South General hospital, two were admitted to NICU but was not in need of NICU procedures. One of these infants was preterm born at gestational week 33 with a mild transient tachypnea and the second patient, a term born baby with a dermatological disease requiring ointment treatment but with no need of other support. None of these newborns showed an LDH above 600 U/L (432 and 468 respectively). The remaining 60 patients were all healthy and discharged as “healthy baby examined at maternity ward”. Among those, two newborns showed an LDH exceeding the value of 600 U/L (in duplicates 618/804 U/L and 744/798 U/L), one was delivered by vacuum extraction and the other with caesarean section after a failed vacuum extraction, but none of these required NICU care.

In the Vietnamese setting all newborn patients admitted to the hospital within 36 h post-partum and with a gestational age of at least 32 weeks with suspected or clinical signs of illness in which the physician on call considered that a blood sample was needed according to the referring doctor, were included, none study candidate eligible for inclusion was missed during the study period. One patient was admitted from home while the rest of the patients were referred from other children’s hospitals or obstetrical units. Among Hanoi patients, there were 14 patients in need of NICU care with ventilator treatment because of neonatal lung disease (respiratory distress and prematurity, n = 3; pneumonia or septicaemia, n = 4; primary pulmonary hypertension, n = 2, respiratory distress and/or meconium aspiration syndrome, n = 5). Three of these patients died during the study period, LDH at admission of these infants were 1028, 929 and 1525 U/L respectively (Table 1). Twelve patients were admitted to standard paediatric care but did not fulfill the criteria for need of NICU. There was a significant difference in term patients showing a high LDH activity (≥900 U/L) in infants in need of NICU (8/11 term patients) compared to patients not in need of NICU (3/12) during first four days after admission (‘N-1’ Chi squared = 5.01, P = 0.03), giving a sensitivity, specificity, positive and negative predictive value using LDH-POC ≥ 900 U/L for prediction of “need of intensive care” of 73, 75, 73 and 75 percent respectively.

**Table 1:** Patients in need of NICU care presented with diagnosis (RDS respiratory distress syndrome, PPHN, persisting pulmonary hypertension, MAS, meconium aspiration syndrome), reference laboratory LDH results, replicates with the LDH-POC test and bias. The BIAS in column 5 is the difference between the reference laboratory values, only measured once, and the individual POC values. Three patients died during the study period (*).

Patient #/sample #	Diagnosis	Reference-Laboratory LDH (U/L)	POC-LDH (U/L)	BIAS
P3.1	Pneumonia	896	805	−91
P3.2			879	−16.8
P5.2	Septic shock*	1373	1028	−344.8
P5.3			1002	−370.4
P10.1	Asphyxia, RDS, pneumonia	1368	1626	258.5
P10.2			1521	153.8
P10.3			2031	663.3
P11.1	RDS, 34w prematurity	730	692	−37.6
P13.1	RDS, pneumonia	875	765	−109.4
P13.2			884	10.2
P15.1	RDS, 33w prematurity	768	716	−51.2
P15.2			713	−53.8
P15.3			672	−95.5
P16.2	PPHN	742.9	956	213.5
P18.3	RDS	1070	1051	−18.5
P21.3	RDS	912	815	−96.9
P22.1	RDS, MAS	1484	1250	−233.1
P22.3			1089	−394.2
P30.1	Pneumothorax, RDS	813	1039	226.7
P30.3			1013	200
P36.1	Asphyxia, RDS*	886	929	43.3
P36.2			872	−13.9
P38.3	PPHN		1225	362.9
P40.1	MAS, PPHN, 33w prematurity*	1553	1525	−27.5
P40.3			1427	−125.1

## Discussion

Timely screening, treatment and management can prevent nearly 85% of the neonatal deaths caused by infections^15,16^. However in low- and middle-income countries, this is often not possible because of challenges faced by the healthcare systems. Distance to health facilities, lack of trained personnel, and inadequate infrastructure can all delay diagnosis. This can in turn result in deaths that may otherwise have been preventable. Implementation of POCTs, defined as tests performed near to or at the site of patient care, enable immediate decision and changes in patient care. Such timely screening and diagnosis can save countless lives. Nonetheless, owing to the complexity of neonatal screening, there is need for simple, affordable, standardized, testing equipment that can generate sensitive and specific results using small blood volumes. The method must be user-friendly, rapid, equipment-free and easily accessible to those in need. Previous research on newborn infants has shown that LDH levels may provide an early predictor of many reasons for serious illness^8^ and therefor may serve as an important complement to clinical examination.

In this work, we developed a new POC technology for LDH analysis, and evaluated the feasibility of using the method in clinical settings for newborn diagnostics. It was based on an easy-to-use consumable device together with a smartphone app, enabling results to be presented within minutes at the POC. Our data showed that the colorimetric assay was highly sensitive and could readily detect all clinically relevant LDH levels. We tested if the POC diagnostics platform, especially designed for use in newborns, would have suitable clinical performance for neonatal settings. Results in this proof of concept study using an early non-commercially available prototype in samples collected at two different clinical settings showed that LDH values measured in minutes with the POC were comparable with standardized laboratory analyses. The fact that the POC method enables haemolysis free plasma separation from whole blood of varying haematocrit concentrations is in particularly important for applications in neonatal diagnostics. Most existing POC devices were designed for adult samples. However, the haemoglobin (Hb), red blood cell (RBC), and white blood cell (WBC) counts in newborns are significantly different from those in older children and adults. Neonatal RBC counts are elevated at birth but during the first postnatal days, there is a marked decline in the production of RBCs, and consequently in Hb concentration. Therefore, POCTs need to be able to handle a broad range of haematocrit. The fact that LDH is a biomarker that leaks out from damaged or stressed cells makes haemolysis free plasma separation a prerequisite for any POC device.

This study is obviously not designed for estimating the clinical and analytical performance of a commercially available POC instrument. Instead the aim was to describe a possible method for a future diagnostic tool fulfilling the ASSURED criteria. LDH is a well-known marker of cell damage in research settings but still rather unexplored as a marker of critical illness in newborns. During the first week of life this enzyme shows a temporal pattern with a peak value seen at approximately 48 h after birth, and also preterm infants show a decreased enzyme activity compared to term babies in the few studies on the subject^8,17–19^. In the clinical part of the study, there was a large difference in age and hours between first sample and first symptom of illness. Still, LDH shows potential as a predictor of NICU need. As estimated, before starting the clinical part of the study, there were a high number of errors due to manual early phase manufacturing of the plastic cartridges used in the study.

In total 121 tests using umbilical cord blood were performed. The anticipated device errors were seen in 26 (21%). In addition, 19 (16%) tests were excluded due to preanalytical errors (sample haemolysis (n = 13) or not enough sample for laboratory analysis (n = 6). In addition the camera did not focus, i.e. creating a blurry picture not suitable for analysis (n = 2), the clinical operator had problems adding sample (n = 2), software problems (n = 5) and others (n = 6). Therefore, a total of 62 tests were considered valid results.

In the Hanoi neonatal ward setting, 49 out of 123 tests from 43 patients were considered valid while 74 were excluded. The reason for exclusion were the anticipated errors in 58 test (31 tests had the detection filter not completely filled due to sample volume variation, plastics malfunctioning or wrong judgment on the activating time; 13 tests had problems on the size of detection and separation filters, due to manual cutting; and 14 tests showed filter patchiness, maybe due to printing/cutting or aging). The remaining 16 excluded tests had levels of bilirubin higher than the detection limit of the device (≥100 µmol/L). One test with LDH > 3000 U/L was also excluded since it was out of our detection range for the reference laboratory. One patient data had to be withdrawn just after samples been taken due to death before the test could be performed.

The number of excluded test results was high. The reason for excluded tests was mainly due to the manual way of manufacturing the consumables used in the study. To coat chemistry on filters, assemble a filter structure suitable for blood separation in low-volume in plastic components manually is obviously not optimal for such a device in clinical use. Still it is of interest and great value to test early phase prototypes in the clinical setting. The fact that clinicians in a stressful environment collaborate and gives feedback on future technology even in the prototype phase enables future value creation for patients as well as health-care workers.

The simplicity of the operation, and the smartphone-based readout makes this POC platform especially attractive for application in resource-limited settings. The smartphone gives real-time output of the colorimetric test that is completely self-contained with data analysis performed by the mobile application. The smartphone can also be interfaced directly with an external computer and mobile communications technology facilitates information management. Finally, the POC system is made relatively cheaply and as a disposable single-use device. While the current prototype is manually produced, the simplicity of the plastic cartridge and low costs of the filters and chemicals make the test quite affordable. Moreover, we believe that scaling up to automated production will reduce significantly the final costs of the tests up to approximately 0.25 euro. In conclusion, we have developed and tested an early phase LDH-POC device for neonatal diagnostics that is affordable, sensitive, specific, user-friendly, rapid and robust, require minimum equipment, and is deliverable to end-users – in short ASSURED.

## Methods

### Principle

The system takes less than 100 µL of whole blood as an input and delivers a measurement of the LDH concentration within four minutes via the app. Figure 1A shows the device. A set of filter papers pre-impregnated with two different chemical reagents coated with a Biodot Frontline HR Contact dispenser (BioDot Limited, Norton Chichester West Sussex, UK) are assembled inside a 2-cm-diameter plastic cartridge. The disposable has 4 distinct filters packed on top of each other in the following sequence: 2 glass filters responsible for the blood filtration, one membrane filter, responsible for the plasma separation and a filter paper, responsible for the color detection. The pore diameter range of the filters is between 0.3–0.7 µm. The chemical reagents used are NBT (nitro blue tetrazolium) and mPMS (1-Methoxy-5-methylphenazinium methyl sulfate). The assay includes also sodium L-lactate and NAD (b-Nicotinamide adenine dinucleotide hydrate). The LDH released in the plasma catalyses the oxidation of lactate to pyruvate with parallel conversion of NAD + to NADH. Subsequent transfer of an electron to mPMS produces mPMSH which reduces NBT to formazan bringing a purple colour to our assay/detection area. Experimentally, blood is applied at an inlet, using a calibrated scientific pipette with a fixed volume of 80 µL, and plasma is separated in less than 15 seconds by capillary force on the paper filters that also transport the plasma onto a chemically impregnated filter. The plasma rehydrates the chemicals, starting a colorimetric reaction, within the visual spectra, that can be detected by a camera in a standard smartphone. After the blood sample is pipetted into the inlet of the device, the cartridge is placed on a designated slot inside a box holding the smartphone (iPhone 4 S, Apple Inc, Cupertino, CA, US) at a fixed distance for automatic imaging. The box had lighting inside to stabilize the lighting conditions between the experiments. The LDH app on the smartphone captures a set of images every 15 seconds and stores the RGB (Red, Green and Blue) values from the images and a text file linked to the system, along with the raw picture, for quantification. To obtain the LDH concentration, the red channel of the RGB image is analysewd. For this, only the parts of the filter which changed the most is considered based on Otsu’s thresholding method^20^ multiple times when comparing two images.

### Pre-clinical testing and calibration

As a first developmental step, the signal (colour development) given in the plastic-cartridge measured with the smartphone camera is correlated with reaction time to investigate the optimal time point for measuring the colorimetric reaction. Thereafter, the signal at that time point is correlated to the LDH reference method (Cobas 8000 c70, Roche Diagnostics, Rotkreuz, Switzerland) at an accredited central hospital laboratory (Karolinska University Hospital, Solna, Sweden). Also, before testing the assay in a clinical setting the signal was tested three times during a 9 weeks period (0, 7 and 9w) to make sure shelf life was appropriate for the clinical part of the study. All these steps were performed on donor blood from expired blood bags spiked with LDH (Lee Biosolutions, Inc, Maryland Heights, MO, US) in four LDH levels of clinical interest (300, 600, 900 and >1500 U/L). Thereafter an algorithm, converting the colour-signal to an LDH value, was calculated and integrated to the software.

All plastic cartridges were manufactured manually including cutting the paper filters and coating them with the chemical assay. Therefore, a number of cartridges had to be discarded when used by clinicians in the clinical part.

### Clinical phase

The study in Sweden was approved by the Ethical Review Board of Stockholm, no 2014/324-31/2 with amendment 2015/539-32, and the study in Vietnam was approved by the Ethical Review Board of Hanoi. The study was registered at Clinical trials.gov Identifier: NCT02379936 and performed after ethical approval from National Hospital of Paediatrics (former name of VNCH) ethical board under control by the Ministry of Health, Vietnam. Informed consent was obtained for study participation and all methods were carried out in accordance with the approved guidelines.

The POC-LDH device was tested in a controlled clinical setting using umbilical cord blood from newborn healthy babies (n = 62). This part of the study was performed from September 29 to October 19, 2015 in the delivery unit at Stockholm South General Hospital, a teaching hospital with around 7,500 deliveries per year. The cord blood was taken with a heparinized syringe immediately after clamping for a routine blood gas analysis, and if left-over, 70–90 µL were used for POC-LDH. Depending on the available amount of blood left in the syringes, both arterial and venous cord blood were tested. All blood samples tested were also sent to a reference laboratory (Clinical Chemistry laboratory at the Stockholm South General Hospital) for measurement of LDH, total bilirubin and haemolysis index, using the same reference methods as used in the pre-clinical phase of the study (Cobas 8000 c70).

Thereafter POC-LDH tests were performed from March 11 to April 23, 2016 at the neonatal unit of Vietnam National Children’s Hospital (VNCH) in Hanoi, an academic hospital with regional responsibility for neonatal intensive care for northern Vietnam, and without a delivery unit. The inclusion criteria were patients admitted to the ward within the first 28 days of life with an ordination of one or multiple blood tests. Parents were then asked for consent and a registration form filled in. When the doctor on call considered a blood test indicated, an extra 300 µl blood for analysis of POC-LDH was added to the total blood volume. The included infants then followed the ordinary clinical routines. An evaluation was done at four days after admission or earlier in case of discharge or death. This follow-up protocol included any neonatal intensive care procedures needed during first four days after admission, e.g. shock-, ventilator- or continuous positive airway pressure (CPAP) – treatment. The physician was blinded to the LDH result. In total, tests analysed with the POC-LDH method were performed in 43 patients (1–3 replicates for each sample) and all tested samples were also sent to Hanoi laboratory for analysis of LDH and bilirubin using a Beckman Counter AU 2700 s/n 9102080 and a Beckman AU 680 s/n 2014043151 system.

In order to investigate if the two central laboratories used as reference methods were comparable in terms of LDH results, two frozen plasma LDH controls were analysed simultaneously in duplicates. A bias <10 percent was considered as comparable.

### Statistical methods

Pearson’s correlation test for non-parametric data was performed using IBM SPSS statistics 21. An umbilical cord LDH value > 600 U/L was considered as pathologically increased^21^ directly after birth while a LDH value > 900 U/L was considered as an pathological elevation in venous samples in term infants included in the Vietnamese setting^8^. If multiple tests were performed from a sample of an included infant, the first value was used for calculating clinical predictive value (sensitivity, specificity, positive and negative predictive value). The Vietnam patients in need of neonatal intensive care (NICU) were compared with the group of patients not in need of NICU procedures together with LDH 900 U/L or more vs. less than 900 U/L using a N-1 Chi Square test for two-by-two tables. Due to the difference in LDH activity in preterm and term infants, preterm patients were not included in this calculation^8^.

## Supplementary information

Sup.Fig.1

## References

[1] WHO|7000 newborns die every day, despite steady decrease in under-five mortality, new report says. *WHO* Available at, http://www.who.int/news-room/detail/19-10-2017-7-000-newborns-die-every-day-despite-steady-decrease-in-under-five-mortality-new-report-says. (Accessed: 17th April 2018).

[2] WHO|Newborn death and illness. *WHO* Available at, http://www.who.int/pmnch/media/press_materials/fs/fs_newborndealth_illness/en/. (Accessed: 17th April 2018).

[3] Liu L (2012). Global, regional, and national causes of child mortality: an updated systematic analysis for 2010 with time trends since 2000. The Lancet.

[4] Mwaniki MK, Baya EJ, Mwangi-Powell F, Sidebotham P (2016). Tweaking’the model for understanding and preventing maternal and neonatal morbidity and mortality in Low Income Countries: inserting new ideas into a timeless wine skin. BMC Pregnancy Childbirth.

[5] Majors CE, Smith CA, Natoli ME, Kundrod KA, Richards-Kortum R (2017). Point-of-care diagnostics to improve maternal and neonatal health in low-resource settings. Lab. Chip.

[6] Thoresen M (2012). Lactate dehydrogenase in hypothermia-treated newborn infants with hypoxic-ischaemic encephalopathy. Acta Paediatr..

[7] Yum SK, Moon C-J, Youn Y-A, Sung IK (2017). Changes in lactate dehydrogenase are associated with central gray matter lesions in newborns with hypoxic-ischemic encephalopathy. J. Matern. Fetal Neonatal Med..

[8] Karlsson M (2012). Lactate dehydrogenase as an indicator of severe illness in neonatal intensive care patients: a longitudinal cohort study. Acta Paediatr..

[9] Ozkiraz S (2013). Lactate and lactate dehydrogenase in predicting the severity of transient tachypnea of the newborn. J. Matern. Fetal Neonatal Med..

[10] Morini F (2008). Lactate dehydrogenase activity is increased in plasma of infants with advanced necrotizing enterocolitis. Pediatr. Surg. Int..

[11] Mabey D, Peeling RW, Ustianowski A, Perkins MD (2004). Tropical infectious diseases: diagnostics for the developing world. Nat. Rev. Microbiol..

[12] Peeling RW, Mabey D, Herring A, Hook EW (2006). Why do we need quality-assured diagnostic tests for sexually transmitted infections?. Nat. Rev. Microbiol..

[13] Kannan B (2015). Printed paper sensors for serum lactate dehydrogenase using pullulan-based inks to immobilize reagents. Anal. Chem..

[14] Banerjee I (2017). Slipdisc: a versatile sample preparation platform for point of care diagnostics. RSC Adv.

[15] Knippenberg R (2005). Systematic scaling up of neonatal care in countries. The Lancet.

[16] Edmond K, Zaidi A (2010). New approaches to preventing, diagnosing, and treating neonatal sepsis. PLoS Med..

[17] Lackmann GM, Töllner U, Mader R (1993). Serum enzyme activities in full-term asphyxiated and healthy newborns: enzyme kinetics during the first 144 hours of life. Enzyme Protein.

[18] Lackmann GM, Töllner U (1995). The predictive value of elevation in specific serum enzymes for subsequent development of hypoxic-ischemic encephalopathy or intraventricular hemorrhage in full-term and premature asphyxiated newborns. Neuropediatrics.

[19] Zanardo V, Bondio M, Perini G, Temporin GF (1985). Serum glutamic-oxaloacetic transaminase and glutamic-pyruvic transaminase activity in premature and full-term asphyxiated newborns. Neonatology.

[20] Otsu N (1979). A threshold selection method from gray-level histograms. IEEE Trans. Syst. Man Cybern..

[21] Wiberg-Itzel E, Josephson H, Wiberg N, Olson L, Winbladh B (2015). Lactic Dehydrogenase in Umbilical Cord Blood in Healthy Infants after Different Modes of Delivery. J Neonatal Biol.

