# Supplementary material for: A rapid smartphone-based lactate dehydrogenase test for neonatal diagnostics at the point of care
Source: Sci Rep. 2019 Jun 26;9:9301. doi: 10.1038/s41598-019-45606-0 (PMC6595069; doi:10.1038/s41598-019-45606-0)
Supplement: Supplementary file 1 — Sup.Fig.1 [file 41598_2019_45606_MOESM1_ESM.docx]

**Supplementary Figures**

**A rapid smartphone-based lactate dehydrogenase test for neonatal diagnostics at the point of care**

Cecilia Pegelow Halvorsen^1,2^, Linus Olson^3,4,5^, Ana Catarina Araújo^6^, Mathias Karlsson^6,7^, Trang Thị Nguyễn^8,9^, Dung T K Khu^5,8^, Ha TT Le^8,9^, Hoa TB Nguyễn^8,9^, Birger Winbladh^1^, Aman Russom^10*^

* Address correspondence to this author at: aman.russom@scilifelab.se


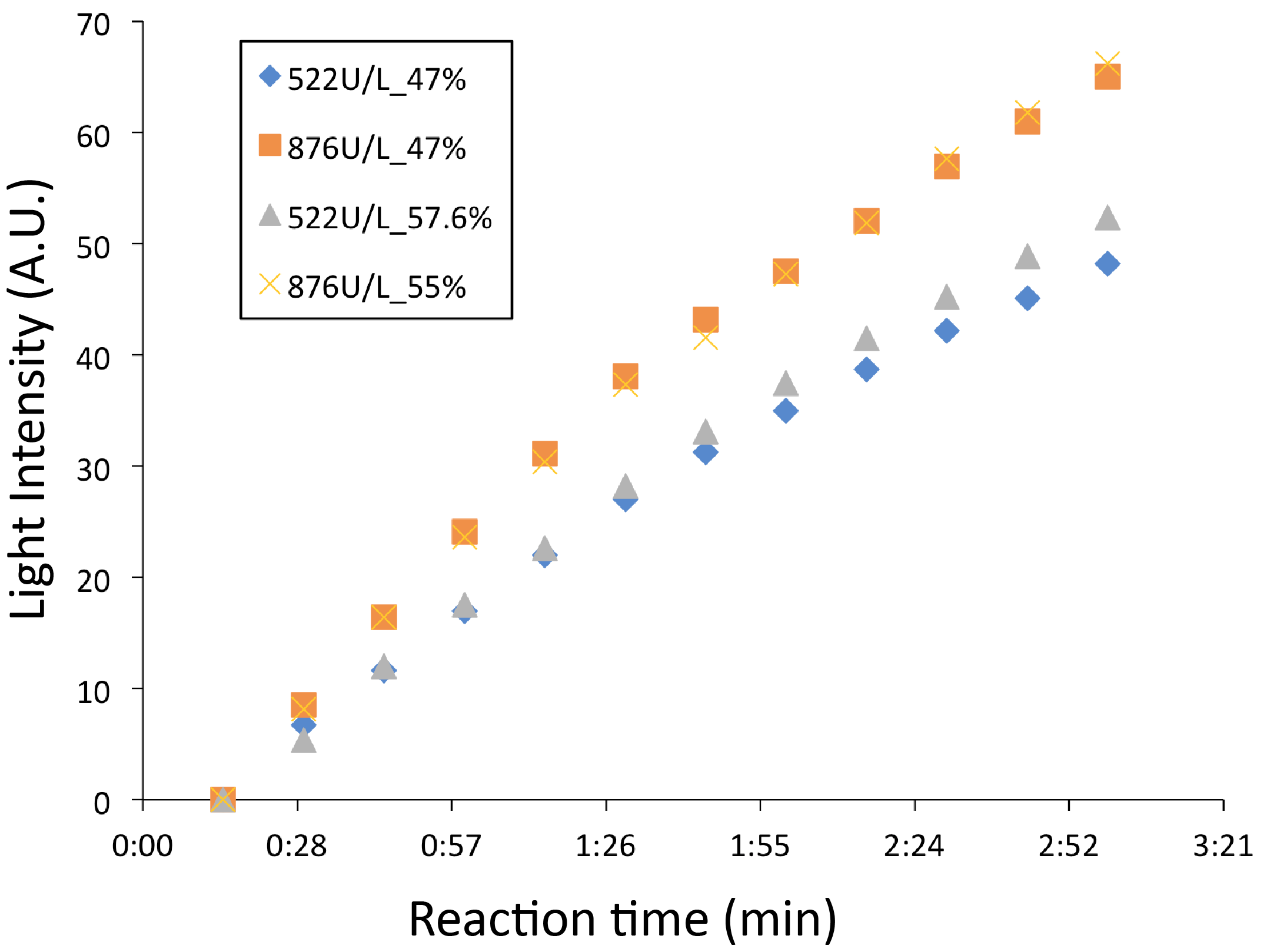


**Sup. Fig.1** Hemolysis-free blood filtration using the POC device. The color development of the LDH assay (light intensity over reaction time) reveals the color intensity is largely independent on the hematocrit concentration, indicating that the plasma separation is hemolysis free.
